# Human Norovirus Triggers Primary B Cell Immune Activation *In Vitro*

**DOI:** 10.1128/mbio.00175-22

**Published:** 2022-04-11

**Authors:** Carmen Mirabelli, Melissa K. Jones, Vivienne L. Young, Abimbola O. Kolawole, Irene Owusu, Mengrou Shan, Basel Abuaita, Holly Turula, Jose G. Trevino, Irina Grigorova, Steven K. Lundy, Costas A. Lyssiotis, Vernon K. Ward, Stephanie M. Karst, Christiane E. Wobus

**Affiliations:** a Department of Microbiology and Immunology, University of Michigan, Ann Arbor, Michigan, USA; b Department of Molecular Genetics and Microbiology, College of Medicine, University of Florida, Gainesville, Florida, USA; c Department of Microbiology and Immunology, School of Biomedical Sciences, University of Otago, Dunedin, New Zealand; d West African Center for Cell Biology of Infectious Pathogens, Department of Biochemistry, Cell and Molecular Biology, University of Ghana, Legon, Accra, Ghana; e Department of Molecular and Integrative Physiology, University of Michigan, Ann Arbor, Michigan, USA; f Division of Surgical Oncology, Department of Surgery, Virginia Commonwealth University, Richmond, Virginia, USA; g Division of Rheumatology, Department of Internal Medicine, University of Michigan Medical School, Ann Arbor, Michigan, USA; h Department of Microbiology and Cell Science, IFAS, University of Florida, Gainesville, Florida, USA; i Graduate Program in Immunology, University of Michigan, Ann Arbor, Michigan, USA; Indiana University—Bloomington

**Keywords:** B cells, calicivirus, immune cell activation, nonstructural protein, noroviruses

## Abstract

Human norovirus (HNoV) is a global health and socioeconomic burden, estimated to infect every individual at least five times during their lifetime. The underlying mechanism for the potential lack of long-term immune protection from HNoV infections is not understood and prompted us to investigate HNoV susceptibility of primary human B cells and its functional impact. Primary B cells isolated from whole blood were infected with HNoV-positive stool samples and harvested at 3 days postinfection (dpi) to assess the viral RNA yield by reverse transcriptase quantitative PCR (RT-qPCR). A 3- to 18-fold increase in the HNoV RNA yield was observed in 50 to 60% of donors. Infection was further confirmed in B cells derived from splenic and lymph node biopsy specimens. Next, we characterized infection of whole-blood-derived B cells by flow cytometry in specific functional B cell subsets (naive CD27^−^ IgD^+^, memory-switched CD27^+^ IgD^−^, memory-unswitched CD27^+^ IgD^+^, and double-negative CD27^−^ IgD^−^ cells). While the susceptibilities of the subsets were similar, changes in the B cell subset distribution upon infection were observed, which were also noted after treatment with HNoV virus-like particles and the predicted recombinant NS1 protein. Importantly, primary B cell stimulation with the predicted recombinant NS1 protein triggered B cell activation and induced metabolic changes. These data demonstrate that primary B cells are susceptible to HNoV infection and suggest that the NS1 protein can alter B cell activation and metabolism *in vitro*, which could have implications for viral pathogenesis and immune responses *in vivo*.

## INTRODUCTION

Human noroviruses (HNoVs) are the most prevalent viruses associated with foodborne illnesses, specifically viral gastroenteritis, which are considered by the World Health Organization to be a major public health concern (1). In addition, the economic burden of HNoV worldwide has been estimated at $60 billion per year (2). HNoVs are from the *Caliciviridae* family, and the circulating strains belong mostly to genogroups GI and GII, with genotype GII.4 being the most prevalent. The development of effective therapeutics has been hampered by HNoV genetic variability (and lack of cross-reactivity) and the historical lack of a culturing system. Despite the ability of HNoV to infect human intestinal enteroids and immortalized B cells (3, 4), no cell culture-derived HNoV stock has been produced yet, and infection is routinely performed with stool samples that are HNoV positive by quantitative PCR (qPCR). For this reason, determinants of HNoV infection *in vivo* are also poorly understood. The uncoating receptor for human norovirus has not been identified yet, although histo-blood group antigen (HBGA) is considered an important attachment factor, as human norovirus susceptibility is linked to secretor status, i.e., the abundance of HBGA as a function of fucosyltransferase 2 (Fut-2). In particular, patients with a nonsense mutation in the Fut-2 gene are resistant to infection, while the overexpression of Fut-2 in human intestinal enteroids improves viral attachment and replication (5). A report of modest viral replication in a line of immortalized B cells, BJAB (4), has opened a controversy in the field regarding whether or not B cells support productive viral replication *in vivo*. Human norovirus antigen was detected in the lamina propria of both humans (in macrophages, dendritic cells, and T cells of biopsy specimens from two immunocompromised patients) (6) and animal models (in macrophages, lymphocytes, and dendritic cells of piglets; dendritic cells of chimpanzees; and cells of the hematopoietic lineage in zebrafish larvae) (7–9) but not specifically or exclusively in B cells. In addition, infection of common variable immunodeficiency patients results in chronic infection and continuous symptomatology, suggesting that immune cell infection is not absolutely required for HNoV susceptibility or induced pathophysiology *in vivo* (10).

On the other hand, it has been estimated that every person experiences HNoV at least five times in their lifetime, suggesting a lack of long-term immune protection (11), but the underlying mechanisms are not understood. Broad protection and its long-term duration are also critical parameters in the development of an effective HNoV vaccine, which is lacking to date (12). B cells are a critical component of effective, long-term immunity. There is therefore a need for targeted studies that explore the relationship between HNoV infection and B cells. In this study, we sought to determine whether primary human B cells support *ex vivo* infection with HNoV and the consequences of infection on B cell functions. Increased HNoV genome levels were observed in primary human B cells derived from blood, spleen, and lymph nodes, which were blocked by the addition of the nucleoside inhibitor 2′‐*C*‐Methylcytidine (2′CMC) or type I interferons (IFNs). Moreover, infection with HNoV but also treatment with HNoV virus-like particles (VLPs) or with the nonstructural protein NS1 affected B cell functional subset distributions over time. Previous work showed that the NS1 protein of the murine and human NoV GI genogroup is secreted from infected or transduced cells, respectively, after caspase 3 cleavage (13). Our data show that NS1-2 from the GII genogroup can also be cleaved *in vitro* and that treatment with the predicted NS1 protein alone induces changes in B cell metabolism, with a strong upregulation of metabolites from the tricarboxylic acid (TCA) cycle, which is consistent with B cell activation. The implications of this finding for HNoV immune responses are potentially manifold and call for more detailed studies.

## RESULTS

### HNoV replicates in primary B cells *in vitro* and is restricted by the type I interferon response.

**FIG 1:**
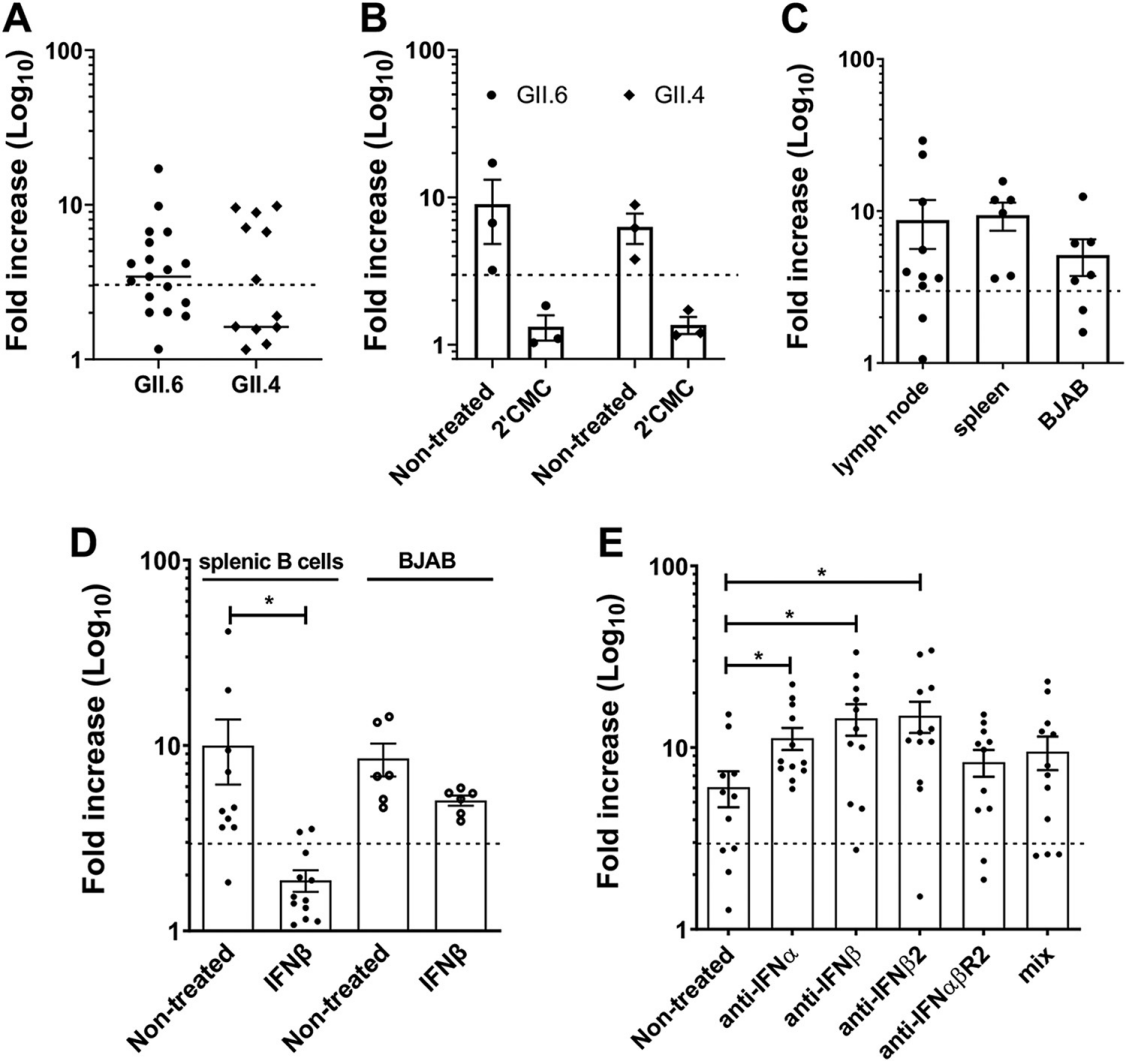
HNoV replicates in primary B cells, and replication is restricted by interferon. (A) Primary B cells were extracted from whole blood and infected with GII.4- and GII.6-positive stool samples in 1.5-mL tubes. One hour after adsorption, cells were washed and incubated for 3 days at 37°C in coculture with hCD40L-3T3 cells. The graph represents the fold increases in viral genome copies obtained by RT-qPCR at 3 dpi versus 0 dpi. Each data point represents a biological replicate (different blood donor). (B) Primary B cells were infected with GII.4- and GII.6-positive stool samples in the absence or presence of 2′-*C*-methylcytidine (2′CMC). The graph represents fold increases in viral genome copies. Each dot is a single biological replicate. (C) Primary B cells were isolated from spleen and lymph nodes of patients’ biopsy specimens. The graph represents the fold increase in viral genome copies. Each dot is a single biological replicate (single donor), which is the average from three technical replicates. (D) Primary B cells from spleen and BJAB cells were treated with IFN-β prior to infection with HNoV-positive stool samples and viral loads determined as before. (E) Primary B cells from spleen were treated with anti-IFN antibodies prior to and for the duration of infection with HNoV-positive stool specimens and viral loads determined as before. For all experiments, each dot represents a technical replicate of infection. The dashed line represents the threshold of a 3-FI, indicative of viral replication. Statistical tests were performed using GraphPad Prism, and Student’s *t* test was performed for all panels. *, *P* value of <0.05.

To determine whether primary human B cells were susceptible to HNoV infection, peripheral blood mononuclear cells (PBMCs) were obtained from the blood of deidentified donors after Ficoll centrifugation. B cells were isolated by using magnetic beads coupled to anti-CD19. The B cells were cocultured with γ-irradiated human CD40 ligand (hCD40L)-expressing 3T3 cells (hCD40-3T3 cells) for 2 days and subsequently infected with GII.4 or GII.6 HNoV-positive stool samples. At 3 days postinfection (dpi), viral RNA was measured by reverse transcriptase qPCR (RT-qPCR), and increases in viral replication were calculated as the fold increase (FI) versus 0 dpi (inoculum). Primary B cells derived from 6/12 (50%) and 11/18 (60%) donors were permissive to replication with GII.4 and GII.6 HNoVs, respectively, with FIs of ≥3 (Fig. 1A). A threshold of a 3-FI was previously defined as an indicator of HNoV replication *in vitro* (14). No HNoV replication was seen in hCD40L-expressing 3T3 cells alone (see Fig. S1A in the supplemental material). Importantly, upon treatment with the nucleoside analog 2′CMC, an inhibitor of the NoV RNA-dependent RNA polymerase, no increase in viral RNA was detected in any of the donors tested (Fig. 1B), suggesting that HNoV actively replicates in primary B cells. To confirm this finding, primary B cells were isolated from human spleen and lymph node biopsy specimens with an EasySep human B cell isolation kit (StemCell Technologies) and infected with GII.6 HNoV-positive stool samples. BJAB, a clone of immortalized B cells that was previously described to support HNoV infection (4), served as a control. B cells from both spleen and lymph node supported HNoV replication, with 80% (8/10) of donors being permissive to infection in the case of lymph node B cells and 100% (6/6) of donors being permissive in the case of splenic B cells (Fig. 1C). Since HNoV infection of intestinal epithelial cells is restricted by interferons (IFNs) (15), we next tested whether HNoV infection of primary B cells was similarly susceptible to type I IFNs. Primary splenic B cells and BJAB cells were treated with IFN-β (1,000 U/mL) for 24 h prior to infection. The treatment reduced the infection by 8-fold in primary B cells but only 3-fold in BJAB cells, suggesting that primary B cells are more sensitive to IFN treatment than BJAB cells (Fig. 1D). Conversely, when primary B cells from spleen tissues were pretreated for 18 h with antibodies neutralizing IFN-α (1:4,000), IFN-β (1:4,000), IFN-β2 (1:4,000), the type I IFN-αβ receptor (1:1,000), or a combination of antibodies (at the concentrations indicated above), HNoV infection increased at least 2-fold (Fig. 1E). Together, these data suggest that primary B cells can support modest HNoV replication *ex vivo*, indicative of abortive infection, and that infection is sensitive to the nucleoside analog 2′CMC and the antiviral activity of type I IFNs.

10.1128/mbio.00175-22.1 FIG S1 (A) Fold increase in viral genome copies of HNoV GII.4-infected hCD40L-expressing 3T3 fibroblasts at 1 and 3 dpi. (B) Graphs of fold increases in viral genome copies over time of culture for two representative donors (D10 and D54). (C) Correlation analysis between fold increases in viral replication and baseline levels of expression of galactoside 2-α-l-fucosyltransferase 2 (Fut-2) and interferon beta (IFN-β). mRNAs for Fut-2 and IFN-β were quantified by qPCR on the different donor samples at 0 dpi (baseline expression). Transcripts are expressed as arbitrary units normalized by the internal control GAPDH and the sample with the lowest FI in replication. Curves were plotted and linear regression was calculated using GraphPad Prism. Every dot represents an individual donor. (D) Representative flow plots of uninfected primary B cells after staining with Live/Dead fixable aqua dead cell stain, PerCP/Cy5.5 anti-human CD20, PE-CF594 mouse anti-human CD27, and Pacific Blue anti-human IgD. Cells were analyzed with a BD Fortessa instrument, and data were plotted with FlowJo software. Download FIG S1, TIF file, 1.3 MB. Copyright © 2022 Mirabelli et al. 2022 Mirabelli et al. https://creativecommons.org/licenses/by/4.0/ This content is distributed under the terms of the Creative Commons Attribution 4.0 International license.

### HNoV infection efficacy in blood-derived B cells is dependent on donor and culturing time.

**FIG 2:**
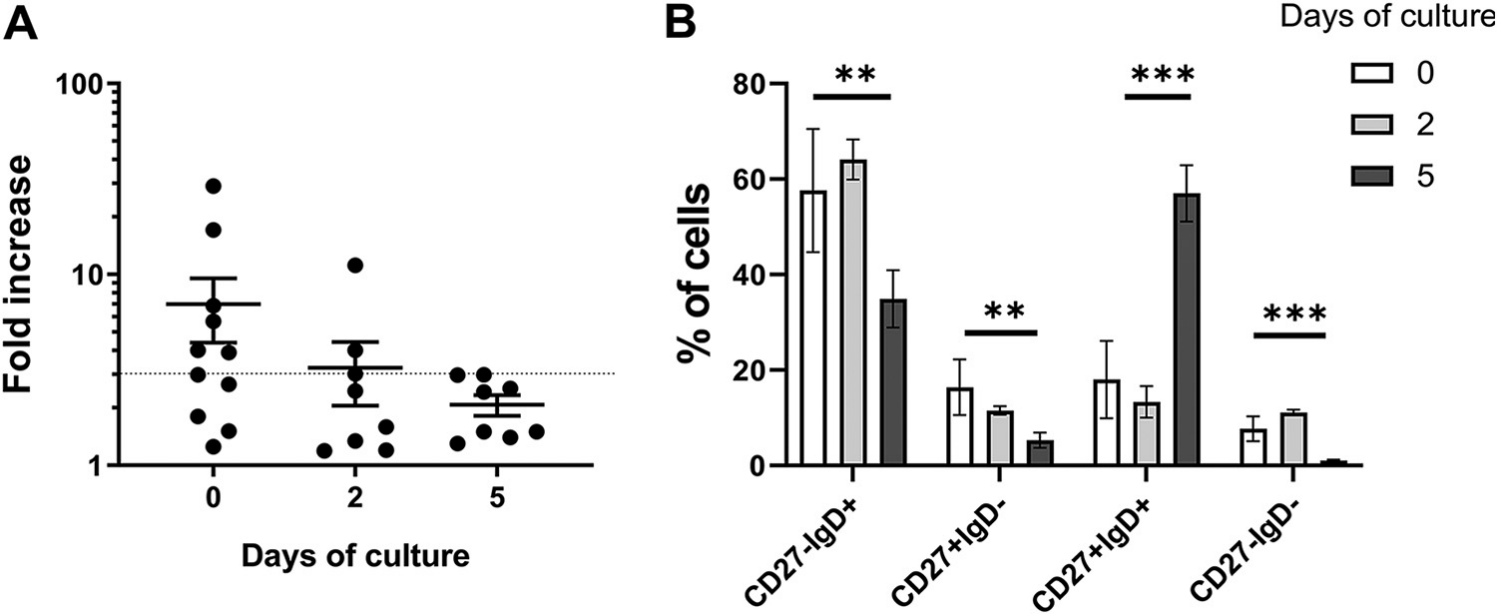
HNoV infection is more efficient in freshly isolated B cells. (A) Whole-blood-derived B cells were infected with GII.6 HNoV-positive stool samples at the moment of isolation or after 2 or 5 days of coculture with hCD40L-3T3 cells. The graph represents the fold increases in viral genome copies obtained by RT-qPCR at 3 dpi versus 0 dpi. Cell viability was determined on uninfected cells by flow cytometry as a percentage of live/dead cells after staining with Live/Dead fixable aqua dead cell stain. Each data point in the graph indicates a biological replicate from one donor (day 0, *n* = 11; day 2 and day 5, *n* = 8). Statistical tests were performed using GraphPad Prism, and the day 5 group is statistically different from the day 0 group by a Mann-Whitney test (*P* = 0.035). (B) Primary B cells from various donors were subjected to flow cytometry analysis after isolation (day 0) (*n* = 10) and at day 2 (*n* = 5) and day 5 (*n* = 5) of coculture with hCD40L-3T3 cells. The percentages of CD27^−^ IgD^+^ (naive), CD27^+^ IgD^−^ (memory-switched), CD27^+^ IgD^+^ (memory-unswitched), and CD27^−^ IgD^−^ (double-negative) B cells were calculated with FlowJo software and represent averages and standard deviations (SD) from 5 to 10 independent experiments. Statistical tests were performed using GraphPad Prism (**, *P* < 0.001; ***, *P* < 0.0001 [according to Student’s *t* test]).

To test whether the efficacy of HNoV infection could be improved in primary B cells, B cells were isolated from whole blood of different donors and infected directly after isolation or cocultured with γ-irradiated hCD40L-expressing 3T3 cells for 2 and 5 days before infection. Infection with GII.6-positive stool samples was more efficient in freshly isolated B cells than in primary B cells in culture for 2 or 5 days, although cell viability did not change over time (82%, 85%, and 83% on days 0, 2, and 5, respectively) (Fig. 2A). Even in HNoV-infected B cells isolated from the same donor, the infection efficiency decreased over the time of culture (Fig. S1B). Given the variability across donors, we sought to establish whether a correlation existed between HNoV infection status and the levels of two previously described determinants of viral replication: fucosyltransferase 2 (Fut-2), an HNoV susceptibility factor, and IFN-β, a restriction factor for HNoV infection (Fig. S1C). mRNA levels were determined by qPCR on the donor samples at 0 dpi, and transcripts were normalized to the internal control glyceraldehyde-3-phosphate dehydrogenase (GAPDH). However, no statistically significant correlation between fold increases in viral replication and IFN-β expression or Fut-2 expression was observed. Therefore, these two known determinants of infection did not explain the differential susceptibilities observed among donors, and future studies will be required to address this point.

Next, we wanted to characterize HNoV infection in primary B cells in more detail. To that end, a flow cytometry pipeline was established to identify functional B cell subsets according to the CD27 marker of memory and IgD expression on the cell surface: naive (CD27^−^ IgD^+^), memory-switched (CD27^+^ IgD^−^), memory-unswitched (CD27^+^ IgD^+^), and double-negative (CD27^−^ IgD^−^) B cells (Fig. S1D). The prevalence of each subset was first determined in noninfected primary B cells freshly isolated or cultured on hCD40L-3T3 cells for 2 and 5 days. A change in the subset distribution was consistently observed across donors (Fig. 2B). The concomitant decrease of HNoV replication and loss of the double-negative and switched memory B cell subsets over time raised the possibility that HNoV selectively infects this specific functional B cell subset(s).

### HNoV tropism is not restricted to a specific B cell subset.

**FIG 3:**
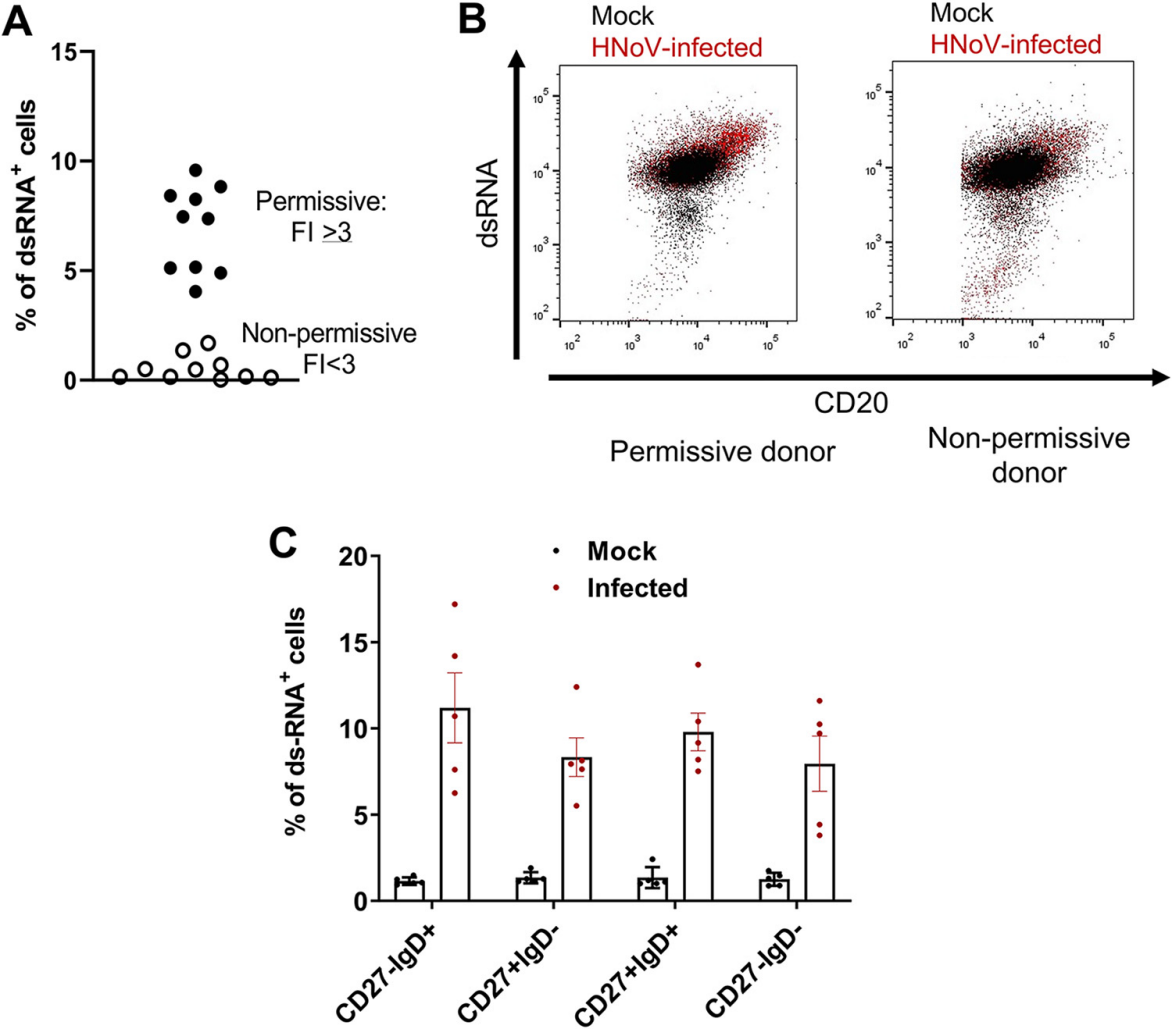
HNoV tropism is not restricted to a specific B cell subset. (A) Freshly isolated primary B cells were infected or mock infected with GII.6-positive stool samples and harvested at 3 dpi for flow cytometry analysis after staining with Live/Dead fixable aqua dead cell stain, PerCP/Cy5.5 anti-human CD20, PE-CF594 mouse anti-human CD27, Pacific Blue anti-human IgD, and biotinylated dsRNA (J2) for the detection of infected cells. B cells were also infected in parallel to determine the fold increase (FI) in viral replication by RT-qPCR. The graph shows the percentage of dsRNA-positive (dsRNA^+^) cells of infected B cells. Each data point is an independent biological replicate from a different donor. Permissive donors were defined according to a 3-FI or higher as defined by RT-qPCR. (B) Representative flow plots of infected and mock-infected B cells from permissive and nonpermissive donors. (C) Percentages of dsRNA^+^ cells in the different functional B cell subsets. The dsRNA^+^ gate was arbitrarily defined on the HNoV-infected B cells from nonpermissive donors to include ≤1% events. Percentages are calculated in the infected gate (4 to 10% of total B cells). Each data point on the graph represents an independent biological replicate from 5 different permissive donors. Averages and SD are also depicted.

To test this hypothesis, freshly isolated blood-derived primary B cells were infected with HNoV GII.6-positive stool samples and subjected to flow cytometry analysis at 3 dpi. Cells replicating the HNoV genome were detected using an antibody against double-stranded RNA (dsRNA), an intermediate of viral replication. HNoV-infected cells ranged between 5 and 10% of the total B cells in donors that were permissive to infection by RT-qPCR (FI > 3), whereas a lower percentage of infected cells corresponded to nonpermissive donors (FI < 3) (Fig. 3A). A representative flow plot of one permissive and one nonpermissive donor is shown in Fig. 3B. However, the proportions of HNoV-infected cells did not differ across the functional B cell subsets (Fig. 3C), suggesting that HNoV tropism is not restricted to one of the B cell subsets that are included in our analysis.

### HNoV infection or treatment with selected viral proteins induces changes in the functional B cell subset distribution.

**FIG 4:**
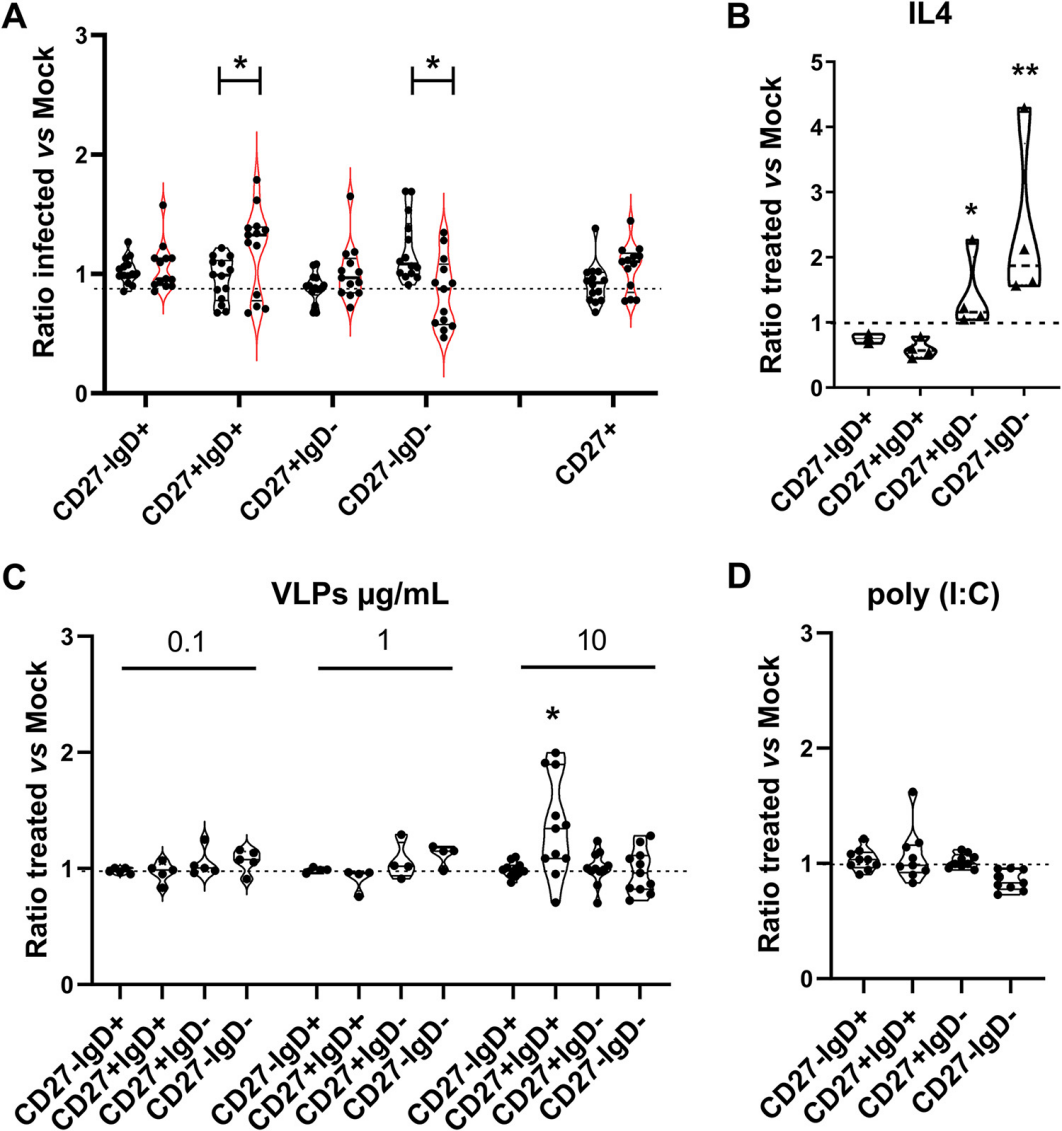
HNoV infection or treatment with virus-like particles (VLPs) induces changes in B cell functional subset distributions. (A) Changes in B cell subsets expressed as a ratio of the percentages in HNoV-infected to noninfected (Mock) B cells. Permissive (*n* = 13) and nonpermissive (*n* = 13) donors were defined according to FIs in viral replication and/or <2% of dsRNA^+^ cells. Values close to 1 represent no or few changes, and values of >1 or <1 represent increases and decreases in the percentages of cells for each subpopulation, respectively. Statistical tests were performed using GraphPad Prism as Student’s *t* test on the ratio between permissive and nonpermissive donors. (B) Changes in B cell subsets upon treatment with IL-4 (20 ng/mL) for 3 days, expressed as a ratio of treated to nontreated (Mock) B cells. (C and D) Changes in B cell subpopulations 3 days after treatment with HNoV VLPs (0.1, 1, and 10 μg/mL) (C) or poly(I·C) (1 μg) (D), expressed as a ratio of treated to nontreated (Mock) B cells. Violin plots depict means and interquartile ranges, and each dot represents an independent biological replicate (single donor). Statistical tests were performed using GraphPad Prism as Student’s *t* test on the raw cell percentages of treated versus mock-treated cells.

To assess the potential impact of HNoV on B cell function, we first compared the distributions of B cell subsets between permissive and nonpermissive donors upon infection. Briefly, from the percentage of cells in each subset, we calculated the ratio for HNoV-infected to mock (noninfected) conditions, whereby a ratio of 1 represents no changes, a ratio of >1 represents an increase, and a ratio of <1 represents a decrease in the subset distribution. We found that upon HNoV infection, double-negative (CD27^−^ IgD^−^) and unswitched (CD27^+^ IgD^+^) B cells exhibited significant changes in prevalence compared with nonpermissive donors (Fig. 4A). As a technical control for the flow cytometry pipeline and a biological control to determine the extent of these changes in the subset distribution, we treated primary B cells with interleukin 4 (IL-4) (20 ng/mL), a cytokine known to promote class switching *in vitro* (16). As expected, IL-4 treatment resulted in an enrichment of switched and double-negative (IgD^−^) subsets (Fig. 4B), and the magnitude of the changes was comparable to the levels observed during HNoV infection (compare to Fig. 4A). To define the molecular triggers for HNoV-induced changes, primary B cells from different donors were treated with GII.4 HNoV virus-like particles (VLPs) at selected concentrations (0.1, 1, and 10 μg/mL) or with the synthetic dsRNA mimic poly(I·C) (1 μg/mL) to account for an effect of viral attachment or viral replication, respectively. We analyzed the B cell subset distribution at 3 days posttreatment (dpt), consistent with the infection time frame. Treatment with HNoV VLPs at the highest concentration tested, but not with poly(I·C), induced a significant enrichment of the unswitched (CD27^+^ IgD^+^) B cell subset (compare Fig. 4C to Fig. 4D), suggesting that HNoV virion attachment may be one trigger of the observed B cell changes.

### HNoV NS1-2 from genogroup GII is cleaved by caspase 7 *in vitro*.

**FIG 5:**
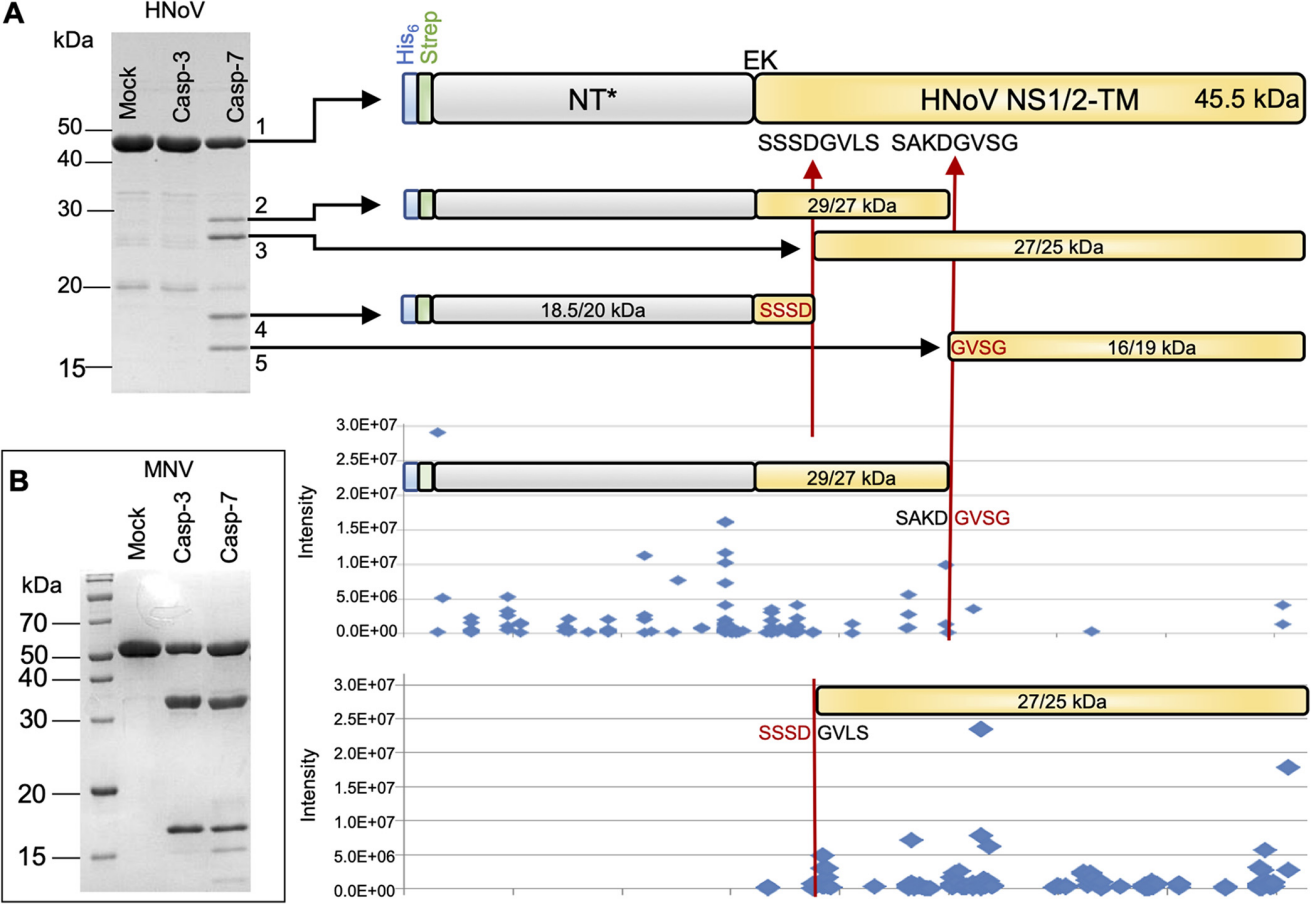
NS1 protein from genogroup GII is cleaved *in vitro* by caspase 7. (A) Fifteen percent SDS-PAGE gel of NT*HNoV NS1/2-TM cleaved with caspase 3 or 7. For the caspase 7 (Casp-7)-treated sample, the uncleaved protein is shown as band 1, and the cleavage products generated by caspase 7 treatment are shown as bands 2 to 5. The identities of protein bands 1 to 5 produced by caspase 7 treatment were confirmed by mass spectrometry and are shown schematically. The protein band schematics for the caspase 7-generated fragments (bands 2 to 5) include the molecular weight (MW) estimated by gel migration and by sequence prediction (gel estimated/predicted MW in kilodaltons). Using mass spectrometry, the C-terminal 4 amino acids of band 4 were determined to be SSSD, and the N-terminal 4 amino acids of band 5 were identified as GVSG, corresponding to two predicted caspase 7 cleavage sites in the full-length protein (SSSDGVLS and SAKGVSG), with the cleavage point indicated by the vertical arrows. The signal intensity of peptides detected by mass spectrometry plotted along the protein sequence supports the expected termini of bands 2 and 3. (B) Fifteen percent SDS-PAGE gel of NT*MNV NS1/2-TM cleaved with caspase 3 or 7. Mock, protein treated in the same way as the caspase samples but without the addition of enzyme.

Previous work showed that NS1-2 of murine norovirus (MNV) and HNoV GI is cleaved by caspase 3 to release NS1 as a secreted protein with the potential ability to alter the bystander cell response to infection (13). However, whether NS1-2 of HNoV GII is also cleaved is unclear. Bioinformatic analysis of HNoV GII NS1-2 using PROSPERous (17) predicted a noncanonical caspase 7 cleavage site (score = 221) at SSSD^26^/GV (P4-P2′) that is also present in MNV and HNoV GI although with a lower rank prediction. To test the ability of caspase 7 to cleave NS1 from HNoV GII, an *in vitro* cleavage assay was performed with caspase 3 used as a control (Fig. 5A). A recombinant baculovirus-expressed HNoV protein from the GII.4 Sydney 2012 strain was expressed and purified. The resulting HNoV NS1/2-TM construct had a deletion of the predicted C-terminal transmembrane (TM) domain but the addition of an N-terminal fusion (NT*) (18) containing a Strep-tag II affinity tag to aid in determining the N terminus after caspase cleavage and SDS-PAGE. The equivalent protein from MNV, NT*.MNV NS1/2-TM (amino acids [aa] 3 to 260), was included as a control (Fig. 5B). Treatment of HNoV NS1/2-TM with caspase 7, but not caspase 3, generated four products that migrated at approximately 29, 27, 18.5, and 16 kDa (Fig. 5A, bands 2, 3, 4, and 5, respectively), suggesting that SSSD^191^/GVLS (18.5- and 27-kDa bands) and SAKD^252^/GVSG (29- and 16-kDa bands) could represent putative caspase 7 cleavage sites. Liquid chromatography (LC)-Orbitrap mass spectrometry (MS) analysis of the 18.5-kDa product (band 4) revealed strong precursor ion intensities for various semichymotryptic peptidoforms with D^191^ (D^26^ without the NT* tag) on the C terminus, indicating the putative caspase cleavage site sequence SSSD^191^ (P4-P1). The 16-kDa cleavage product (band 5) showed strong tryptic peptide signals covering the sequence from G^253^ (G^88^ without the NT* tag) to the protein C terminus by MS analysis. No significant signal was detected N terminally of G^253^, whereas the tryptic digest of the full-length protein (band 1) revealed strong peptide signal intensities throughout the full protein sequence (98% sequence coverage). The peptide signal intensities (intensity plots in Fig. 5A) for the 29- and 27-kDa products (bands 2 and 3), compared to those of the full-length protein, showed that there is a high signal intensity only N terminally of the predicted cleavage site in the 29-kDa band and C terminally of the predicted cleavage site in the 27-kDa band, suggesting that the 29- and 27-kDa bands are N- and C-terminal products, respectively. The Strep-tag II affinity tag was identified in both of the predicted N-terminal caspase-generated proteins (29 kDa and 18.5 kDa) (Fig. 5A, bands 2 and 4) by MS, confirming that both products are derived from the N terminus. Consistent with the bioinformatic prediction, MNV NS1/2-TM showed the same cleaved products after caspase 3 and 7 treatments (Fig. 5B), albeit its cleavage appeared more efficient than that of the HNoV protein, possibly due to the canonical nature of the cleavage site. These data confirm that caspase 7 can cleave NS1-2 to release NS1 and that caspase 7 cleavage might be conserved across genogroups.

### NS1 recombinant protein induces primary B cell activation and metabolic changes.

**FIG 6:**
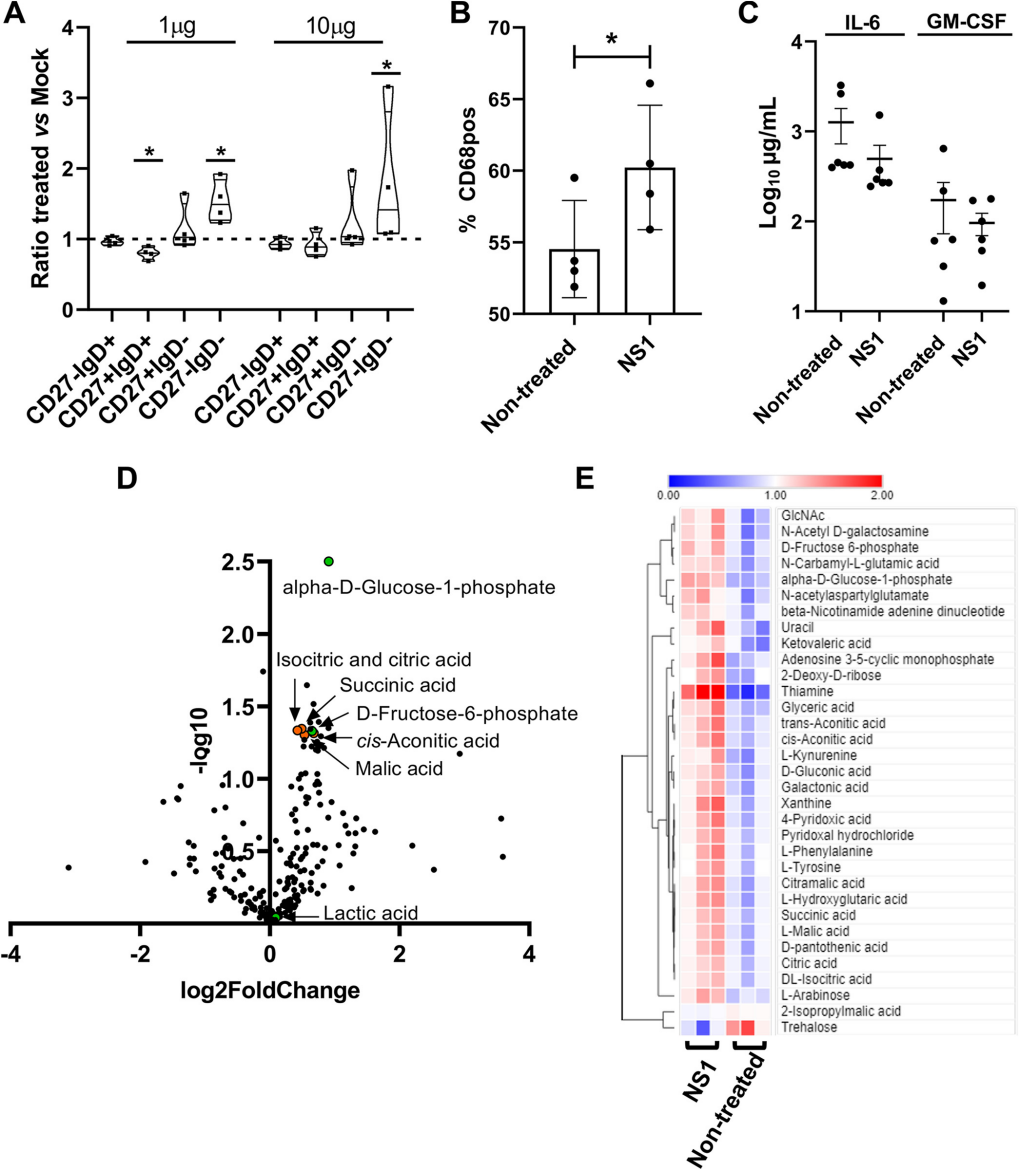
NS1 treatment enhances B cell activation and metabolism. (A) Changes in B cell subpopulations 3 days after HNoV NS1 treatment (1 and 10 μg). (B) Primary B cells were collected and treated with HNoV NS1 (10 μg). Cells were harvested at 3 dpt and prepared for flow cytometry analysis to quantify the surface expression of the activation marker CD68. Cells were analyzed with a BD Fortessa instrument and FlowJo software. Each data point represents a technical replicate from 4 independent biological experiments. (C) Supernatants from primary B cells treated with HNoV NS1 (10 μg) were analyzed by ELISA. (D) A total of 10^6^ primary B cells from three different donors were treated with HNoV NS1 protein (10 μg) for 16 h. Cells were harvested, and metabolites were collected in 80% ice-cold ethanol, followed by snapshot metabolomics analysis. Data are visualized by Volcano plot produced in GraphPad Prism, with the *x* axis representing the log_2_ fold change of selected metabolites under NS1-treated versus nontreated conditions and the *y* axis representing the −log_10_ *P* value for each metabolite. (E) Heat map of metabolites significantly upregulated (red) and downregulated (blue) under NS1 conditions versus nontreated conditions. Data from three technical replicates are shown in the heat map.

To determine whether NS1 can alter B cell features, freshly isolated primary B cells were treated with 1 and 10 μg/mL of the recombinant predicted HNoV GII.4 NS1 protein, and the B cell subset distribution was evaluated by flow cytometry at 3 dpt, as described above. A significant dose-dependent change in unswitched and double-negative B cell subsets was observed (Fig. 6A). Furthermore, an increase in the activation marker CD86 on the surface of primary B cells was observed (Fig. 6B). CD86 is a marker that is upregulated in response to antigens such as lipopolysaccharide (LPS), suggesting that NS1 triggers B cell activation *in vitro*. We also sought to determine whether B cell activation by NS1 was accompanied by changes in the secretion of specific cytokines. We quantified interleukin 6 (IL-6) and granulocyte-macrophage colony-stimulating factor (GM-CSF) in the supernatant of primary B cells 3 days after treatment with recombinant predicted NS1 (10 μg/mL) by an enzyme-linked immunosorbent assay (ELISA) since these two cytokines were significantly upregulated in NS1-stimulated B cells in a Luminex pilot study (data not shown). However, we did not observe statistically significant changes upon NS1 treatment (Fig. 6C). B cell activation is typically associated with changes in B cell metabolism, of which increases in oxidative phosphorylation (OXPHOS), the tricarboxylic acid (TCA) cycle, and nucleotide biosynthesis are hallmarks (19). Thus, we next determined the effect of the NS1 treatment on B cell metabolism by liquid chromatography-coupled tandem mass spectrometry (LC-MS/MS)-based metabolomics analysis (20). Freshly isolated primary B cells from three different donors were stimulated for 16 h at 37°C with 10 μg/mL of recombinant predicted NS1. Analysis of intracellular metabolites showed a strong induction of metabolites of the TCA cycle (citric and isocitric acids, malic acid, succinic acid, and *cis*-aconitic acid) in cells from all donors analyzed in the presence of NS1 (Fig. 6D). In addition, α-d-glucose-1-phosphate and fructose-6-phosphate were two of the most significantly altered metabolites, while the modest increase in lactate is consistent with previously published data on the upregulation of glucose metabolism and the TCA cycle during B cell activation (Fig. 6D and E) (20). Together, these data suggest that the recombinantly expressed predicted NS1 protein induces metabolic and functional changes in B cells that might lead to B cell activation and immune modulation of bystander cells.

## DISCUSSION

In this study, we sought to determine whether human primary B cells are susceptible to HNoV infection *ex vivo* and the potential impact on B cell function. We first demonstrated that HNoV replicates in human B cells derived either from whole-blood PBMCs or from patients’ spleen and lymph node biopsy specimens. Treatment with the nucleoside analog 2′CMC or type I IFN abolished HNoV replication, suggesting that B cells are susceptible and permissive. However, given the low levels of the increases in viral genome titers and dsRNA, HNoV infection of B cells is likely defective, resulting in an abortive infection. Future studies using paired samples from tissue and blood are needed to determine whether the susceptibilities of B cells from different sites are similar or divergent. In addition, among the permissive blood donors, only 5 to 10% of primary blood-derived B cells were positive for viral dsRNA, suggesting that the replication of HNoV is limited to a subset of B cells. However, we could not define this subset based on the memory marker CD27 and IgD, and further studies are needed to define this population after fluorescence-activated cell sorter (FACS) sorting, possibly by means of transcriptome sequencing (RNAseq) or cytometry by time of flight (CyTOF). The low percentage of infected cells in the more homogeneous BJAB model (21) and also in the context of murine norovirus infection in B cells (7 to 9% of primary murine B cells [our unpublished data] and ∼10% of the M12 cell line [4]) suggests that other factors might define the susceptible subset (such as the expression levels of the viral receptor and the metabolic state of the cells, etc.). We analyzed the expression of the antiviral IFN-β and Fut-2, the enzyme required for the addition of terminal fucose to carbohydrate chains, the attachment factor, but no statistically significant correlation with HNoV replication was observed *in vitro*. Therefore, the specific nature of the susceptibility factor(s) in B cells remains to be identified in future studies.

Intriguingly, our study identified the ability of HNoV infection or treatment with the viral protein NS1 or VP1 (VLP treatment) to drive significant changes in B cell functional subsets, especially in the unswitched double-negative compartments. Unswitched cells are defined as B cells that have “seen” the antigen but have not undergone isotype switching that is required to produce antigen-specific antibodies (22). The enrichment of this population together with CD86 expression might be consistent with a model described in the context of infection by other pathogens whereby the induction of polyclonal B cell activation and the production of low-specificity antibody might dilute the pathogen-specific antibody response (23). On the other hand, double-negative B cells are an understudied subset that was recently associated with autoimmune diseases. Interestingly, the prevalence of double-negative cells is higher in rotavirus (RV)-positive memory B cells than in the total memory B cells of vaccinated healthy donors, suggesting specific RV-induced expansion and maintenance of this pool (24). In addition, in HIV-infected patients, the double-negative B cell subset (defined as tissue-like memory B cells in this study) expressed patterns of homing and inhibitory receptors characteristic of T cell exhaustion, suggesting that the enrichment of this population might lead to premature exhaustion and contribute to poor antibody responses during HIV-1 infection (25). More detailed studies on the role of the double-negative B cell subset in the immune response against HNoV infection are required.

We further demonstrated that treatment with the predicted NS1 alone increases CD86 levels and cellular metabolism, features that are consistent with B cell activation. Particularly, we consistently observed across three independent donors a strong metabolic signature of activation of the TCA cycle, a metabolic pathway that has previously been described in activated B cells (20). Whether the replication of HNoV in B cells occurs *in vivo* remains to be resolved. However, the secretion of HNoV NS1 from other infected cell types (e.g., epithelial cells) could stimulate metabolism and in turn activate surrounding B cells. Importantly, a previous study demonstrated NS1 secretion upon caspase 3 cleavage from murine norovirus and the HNoV Norwalk GI genogroup (26). In this study, we used a more clinically relevant GII HNoV and investigated the ability of caspases 3 and 7 to cleave NS1-2 *in vitro*. Interestingly, we found that caspase 7, but not caspase 3, is responsible for GII NS1 cleavage and that the presence of a caspase 7 cleavage site was also conserved in murine norovirus albeit at different locations. This suggests that caspase 7 might be a pan-norovirus caspase that cleaves the NS1-2 protein. However, confirmation of NS1 cleavage by caspase 7 and secretion during HNoV infection requires further investigations.

Collectively, our data suggest a model whereby an enteric virus, through direct infection or bystander effects, induces changes in the activation profile of B cells and possibly other immune cells. Hence, the activation of B cells in the lamina propria or gut-associated lymphoid tissue (GALT) could have profound implications for the development of adaptive immune responses and protection from reinfection. Future studies will be required to test this hypothesis. In conclusion, our study demonstrates that a proportion of primary B cells is susceptible to HNoV infection *in vitro* and highlights a new function for NS1 in B cell activation with possible implications for viral pathogenesis.

## MATERIALS AND METHODS

### Reagents and virus.

2′‐*C*‐Methylcytidine (2′CMC), an *in vitro* inhibitor of HNoV replication, was supplied by Sigma-Aldrich. Human IFN-β was purchased from PBL Assay Science and used at a concentration of 1,000 U/mL. IFN-neutralizing antibodies were obtained from PBL Assay Sciences and were used at the following concentrations: 1:4,000 for anti-IFN-α, 1:4,000 for anti-IFN-β, 1:4,000 for anti-IFN-β2, and 1:1,000 for anti-IFN-αβR2. HNoV GII.4 virus-like particles (VLPs) were purchased from The Native Antigen Company, and poly(I·C) was obtained from InvivoGen (catalog no. tlrl-picwlv). GII.4 Sydney and GII.6 HNoV-positive stool samples were kindly provided by J. Vinje (Centers for Disease Control and Prevention, USA) and S. M. Karst (University of Florida, USA), respectively. Stool samples were diluted with phosphate-buffered saline (PBS) to make a 10% (wt/vol) stock solution. Diluted stool samples were vortexed and centrifuged at 20,000 × *g* for 1 min. The supernatants were used for infection.

### Primary B cell isolation from whole blood.

Thirty-milliliter PBS flowthrough plasmapheresis filters or peripheral blood diluted in PBS (1:3) was collected in 50-mL tubes and underlaid with Ficoll-Paque (GE Healthcare). The blood was obtained from the blood bank at the University of Michigan from deidentified donors. PBMC-containing buffy coat was obtained after centrifugation for 30 min at room temperature at 1,200 relative centrifugal force (RCF). After washing, PBMCs were incubated with anti-CD19 magnetic beads (MACS; Miltenyi Biotec) in MagSep buffer (PBS^−/−^, PBS without Mg2^+^ and Ca2^+^, supplemented with 0.5% bovine serum albumin [BSA] and 2 mM EDTA) for 15 min and washed in MagSep buffer twice before separation by flow on a magnetic column. The B cell fraction obtained after the flowthrough was resuspended in maintenance buffer (Iscove’s modified Dulbecco’s medium [IMDM] supplemented with 10% fetal calf serum, 50 μg/mL of transferrin, a 5-μg/mL mixture of transferrin-insulin-selenium, and 15 μg/mL of gentamicin). Cells were cultured in the presence of previously γ-irradiated hCD40L-3T3 cells (27) at a ratio of 1:8.

### Infection of blood-derived primary B cells with HNoV and viral quantification by RT-qPCR.

Freshly isolated B cells, or B cells that were cocultured with hCD40L-3T3 cells for 2 or 5 days after isolation, were infected with HNoV-positive stool samples of genotype GII.4 or GII.6. Briefly, for freshly isolated B cells, infection occurred in 1.5-mL tubes for 2 h at 37°C, followed by two washes with maintenance medium and seeding in the presence of γ-irradiated hCD40L-3T3 cells. For B cells in culture, HNoV-positive stool samples of genotype GII.4 or GII.6 were spin inoculated for 30 min at 800 × *g* at room temperature, followed by two washes with maintenance medium. One batch of B cells was then harvested immediately after infection (day 0 of infection) with Tri reagent (Zymo Research), and the rest of the infected cells were kept in culture for 3 days at 37°C. Infection was determined by RT-qPCR as the fold increase in viral genome copies at day 3 versus day 0 of infection. Briefly, viral RNA extraction was performed with the Direct-zol RNA MiniPrep Plus kit (Zymogen Research), and HNoV titers were determined by one-step RT-qPCR as previously described (28).

### *Ex vivo* isolation of splenic and lymph node B cells.

Spleen and lymph node tissue samples were collected in compliance with the University of Florida Institutional Review Boards (IRB no. 201600873) and protection of human subjects. Deidentified biopsy specimens of spleen and adjacent lymph node chains, taken during routine clinically indicated operative procedures, were obtained and mashed in 100-μm and 70-μm cell strainers inside a petri dish with 2 mL of PBS supplemented with 2% fetal bovine serum (FBS). Cells were transferred into a 15-mL conical tube and centrifuged for 5 min at 500 × *g*. Germinal cells were resuspended in fresh medium for counting, while splenocytes were incubated in 1 mL of ACK lysis buffer to remove red blood cells (catalog no. A1049201; Thermo Fisher) for 3 min at room temperature and, after centrifugation, were resuspended in fresh medium for counting. After counting, cells were centrifuged at 500 × *g* for 15 min at room temperature and resuspended in RoboSep buffer for B cell isolation by negative selection with an EasySep human B cell isolation kit (StemCell Technologies) according to the manufacturer’s instructions. After isolation, B cells were kept in complete culture medium (RPMI 1640 containing 10% FBS [Omega Scientific] and supplemented with 1× penicillin-streptomycin [Pen/Strep] [Cellgro]), seeded into 48-well plates at a concentration of 1 × 10^5^ cells per well, and incubated 37°C at 5% CO_2_ overnight to 24 h prior to infection.

### Infection of germinal or splenic primary B cells with HNoV.

HNoV-positive stool samples were diluted 1:10 in complete culture medium, and 100 μL of the virus preparation was used to infect cells for 2 h at 37°C at 5% CO_2_. After infection, cells were centrifuged at 750 × *g* for 7.5 min and resuspended in culture medium. Wells for day 0 of infection were immediately harvested in Tri reagent (Zymo Research) for RNA extraction. The remaining wells were incubated at 37°C for 3 days. For treatment with type I IFN, immediately after plating, IFN was added to the wells, and cells were incubated for 24 h prior to HNoV infection. For treatment with anti-IFN antibodies, antibodies were added to wells immediately after plating and incubated with cells for 18 h prior to infection. Medium supplemented with anti-IFN antibodies was used throughout the infection, and medium with antibodies was refreshed daily. Infection was determined by RT-qPCR as the fold increase in viral genome copies at day 3 versus day 0 of infection.

### Flow cytometry analysis for B cell functional subsets.

HNoV-infected or mock-infected primary B cells were harvested at selected times postinfection in MagSep buffer (PBS supplemented with 0.5% BSA and 2 mM EDTA). Cells were first stained with Live/Dead fixable aqua dead cell stain (Thermo Fisher Scientific), and after washing, they were incubated with the surface markers peridinin chlorophyll protein (PerCP)/Cy5.5 anti-human CD20 (catalog no. 302326; BioLegend), phycoerythrin (PE)-CF594 mouse anti-human CD27 (catalog no. 562297; BD Biosciences), and Pacific Blue anti-human IgD (catalog no. 348224; BioLegend). In selected experiments, fluorescein isothiocyanate (FITC) anti-human CD86 (catalog no. 555657; BD Biosciences) was also used. After 20 min, cells were washed and fixed/permeabilized with a fixation/permeabilization solution kit (catalog no. 554714; BD Biosciences) according to the manufacturer’s instructions. Next, cells were stained with an anti-double-stranded RNA (dsRNA) antibody (J2; Scicons), previously biotinylated with the EZ-Link micro N-Hydroxysulfosuccinimide-polyethylene glycol (NHS-PEG4) biotinylation kit (catalog no. 21955; Thermo Fisher) to increase the specificity and sensitivity of the assay, and with an allophycocyanin (APC)/Cy7 streptavidin antibody (catalog no. 405208; BioLegend). Data were acquired with a BD Fortessa instrument and analyzed by using FlowJo. Compensation was performed on uninfected BJAB cells.

### NS1 protein purification.

The HNoV GII.4 (Sydney 2012) (GenBank accession no. JX459908.1) NS1/2-TM (amino acids 3 to 249), MNV-1 (accession no. DQ285629) NS1/2-TM (amino acids 3 to 260), or HNoV GII.4 (Sydney 2012) (accession no. JX459908.1) putative NS1 (amino acids 1 to 134) region was expressed in Trichoplusia ni insect cells using the commercial recombinant baculovirus system Flashback Ultra (Oxford Expression Technologies). The expression construct contained an N-terminal His-Strep-tag II tag, a spidroin NT* solubility tag (18), an enterokinase (EK) cleavage site, and a flexible linker, GGSRS, adjacent to HNoV NS1. Following expression at 27°C for 3 days, the cells were lysed in buffer containing 50 mM NaH_2_PO_4_·2H_2_O (pH 8), 300 mM NaCl, and 10% glycerol with 1% Triton X-100. The protein was purified on Streptactin XT superflow beads (IBA Lifesciences) and eluted with a solution containing 50 mM NaH_2_PO_4_·2H_2_O (pH 8), 300 mM NaCl, and 50 mM biotin. To remove the NT* tag, the HNoV GII.4 NS1 region (aa 1 to 134) was buffer exchanged into enterokinase cleavage buffer (20 mM Tris [pH 8], 50 mM NaCl, 2 mM CaCl_2_) and cleaved using bovine EK (New England BioLabs [NEB]) at 16 U/mg protein for 4 h. The EK was removed using soybean trypsin inhibitor agarose (Sigma), and the cleaved NT* tag was removed using Ni-nitrilotriacetic acid (NTA) resin. The purified HNoV NS1 protein was then buffer exchanged into 20 mM citrate phosphate buffer (pH 6.1)–150 mM NaCl and stored at −80°C.

### *In vitro* caspase cleavage assay.

The MNV NS1/2-TM or HNoV GII.4 (Sydney 2012) NS1/2-TM purified proteins were buffer exchanged using an Amicon Ultra 10,000-molecular-weight-cutoff (MWCO) centrifugal filter and diluted to 1 mg/mL into caspase cleavage buffer {50 mM HEPES (pH 7.4), 100 mM NaCl, 1 mM EDTA, 10 mM dithiothreitol (DTT), 10% glycerol, 0.1% 3-[(3-cholamidopropyl)-dimethylammonio]-1-propanesulfonate (CHAPS)}. Each protein (10 μg) was incubated with either 0 μg or 0.05 μg active human caspase 3 or 0.23 μg active human caspase 7 (Abcam) at either 37°C for 1 h (NT*.MNV NS1/2-TM) or overnight at room temperature (NT*.HNoV NS1/2-TM) to accumulate more of the caspase 7 product for subsequent mass spectrometry analysis. Reactions were stopped by the addition of an equal volume of 2× SDS-PAGE sample buffer, and 5 μg was loaded onto a 15% SDS-PAGE gel. Gels were stained using Coomassie brilliant G250 stain. To identify the protein bands produced upon caspase 7 cleavage of NT*HNoV NS1/2-TM, each of the protein bands was excised in duplicate, including the corresponding full-length non-caspase-cleaved NS1/2-TM from the same lane, as a negative control. Samples were submitted to the Centre for Protein Research, University of Otago, and each band was in-gel digested with either trypsin or chymotrypsin. The resultant peptides were analyzed by a nanoflow-uHPLC (ultrahigh-performance liquid chromatography) system coupled inline to an LTQ Orbitrap XL mass spectrometer (Thermo Scientific) to identify the sequence coverage in the two cleaved protein products in comparison to the full-length protein. Therefore, peptides of each protein digest were separated on an in-house-packed emitter tip column (75-μm-internal-diameter [ID] PicoTip fused silica tubing [New Objectives, Woburn, MA] packed with Aeris 2.6-μm peptide XB-C_18_ material [Phenomenex] on a length of 20 cm) using a gradient developed from 5% mobile phase B (90% acetonitrile, 0.1% formic acid in water) in mobile phase A (1% acetonitrile, 0.1% formic acid in water) to 25% B over 23 min, followed by an increase to 40% B over 5 min and to 99% B over 3 min. Eluting peptides were injected into the nanospray ionization source of the mass spectrometer over the full length of the gradient at a flow rate of 300 nL/min. The Orbitrap mass analyzer was operated in full-MS mode at a resolution of 60,000 at *m/z* 400. The 10 strongest ion signals per cycle were selected for the acquisition of data-dependent collision-induced dissociation (CID) fragment ion spectra. Singly charged precursor ions were excluded. Dynamic exclusion was enabled allowing two repeated acquisitions of CID spectra on the same precursor ion over a period of 90 s.

### Mass spectrometry data analysis.

Raw data were searched against the target sequences integrated into the larger sequence context of the human reference sequences using Proteome Discoverer software (version 2.4; Thermo Scientific). No enzyme specificity was selected for the search, and oxidation of methionine as well as deamidation of asparagine and glutamine were allowed as variable modifications. Carboxyamidomethylated cysteine was selected as a static modification. The “fixed PSM validator” node in Proteome Discoverer software was used to filter significant peptide spectrum identifications at score (Xcorr) thresholds of 2, 2.5, and 3 for charge stages 2, 3, and ≥4, respectively. Only peptide spectrum matches listed as being of high confidence by Proteome Discoverer software were considered.

To visualize the significantly identified sequence coverage of each cleavage product and the full-length protein, the area under the curve of each significantly identified peptide was extracted from the search output and plotted against the position of the first amino acid in the respective peptide along the protein sequence.

### Metabolomics analysis of intracellular metabolites.

Freshly isolated primary B cells (10^6^ cells/well) were cultured with previously γ-irradiated hCD40L-3T3 cells in the presence or absence of NS1 (1 μg/mL) for 16 h at 37°C. After incubation, cells were collected by centrifugation at 500 × *g* for 5 min at 4°C. Cell pellets were resuspended in ice-cold 80% methanol and kept at −80°C for 10 min. The supernatants were then collected after centrifugation at the highest speed for 5 min at 4°C. Metabolites were dried at 4°C using a SpeedVac. Metabolite pellets were reconstituted in 50 μL of 50% methanol, and 40 μL was transferred to an autosampler glass vial for untargeted LC-MS analysis. Samples were run on an Agilent 1290 Infinity II LC-6470 triple-quadrupole (QqQ) tandem mass spectrometry (MS/MS) system with the following parameters. The Agilent Technologies triple-quadrupole 6470 LC-MS/MS system consists of the 1290 Infinity II LC flexible pump (quaternary pump), the 1290 Infinity II multisampler, the 1290 Infinity II multicolumn thermostat with a 6-port valve, and the 6470 triple-quadrupole mass spectrometer. Agilent MassHunter Workstation software LC-MS data acquisition for 6400 series triple-quadrupole MS with version B.08.02 was used for compound optimization, calibration, and data acquisition.

### LC.

Two microliters of the sample was injected into an Agilent Zorbax rapid-resolution high-definition (RRHD) Extend C_18_ column (2.1 by 150 mm, 1.8 μm) with Zorbax Extend fast guards. The LC gradient profile is as follows, using the solvent conditions described below: 100% solvent A at 0.25 mL/min from 0 to 2.5 min, 80% A and 20% B from 2.5 to 7.5 min, 55% A and 45% B from 7.5 min to 13 min, 1% A and 99% B from 13 min to 24 min, 1% A and 99% C from 24 min to 27 min, 1% A and 99% C from 27 min to 27.5 min, 1% A and 99% C at 0.8 mL/min from 27.5 to 31.5 min, 1% A and 99% C at 0.6 mL/min from 31.5 to 32.25 min, 100% A at 0.4 mL/min from 32.25 to 39.9 min, and 100% A at 0.25 mL/min at 40 min. The column temperature was kept at 35°C, and samples were stored at 4°C.

### Solvents.

Solvent A is composed of 97% water and 3% methanol with 15 mM acetic acid and 10 mM tributylamine at pH 5. Solvent B is composed of 15 mM acetic acid and 10 mM tributylamine in methanol. Washing solvent C is composed of acetonitrile. The LC system seal washing solvent is composed of 90% water and 10% isopropanol, and the needle wash solvent is composed of 75% methanol and 25% water. The following solvents were purchased from the indicated vendors: gas chromatography (GC)-grade 99% tributylamine (Acros Organics); LC-MS-grade acetic acid Optima (Fisher Chemical); InfinityLab deactivator additive, ESI-L low-concentration tuning mix (Agilent Technologies); LC-MS-grade solvents of water, acetonitrile, and methanol (Millipore); and isopropanol (Fisher Chemical).

### MS.

The 6470 triple-quadrupole MS system was calibrated with the Agilent ESI-L low-concentration tuning mix. The source parameters were as follows: gas temperature of 150°C, gas flow rate of 10 L/min, nebulizer at 45 lb/in^2^, sheath gas temperature of 325°C, sheath gas flow rate of 12 L/min, capillary at −2,000 V, and delta electron multiplier voltage (EMV) of −200 V. The dynamic multiple-reaction monitoring (MRM) scan type was used with a 0.07-min peak width, and the acquisition time was 24 min. A delta retention time of ±1 min, a fragmentor at 40 eV, and a cell accelerator at 5 eV were incorporated into the method.

### Data analysis.

The MassHunter metabolomics dynamic MRM database and method were used for target identification. Key parameters of Agilent jet stream (AJS) electrospray ionization (ESI) were as follows: gas temperature of 150°C, gas flow rate of 13 L/min, nebulizer at 45 lb/in^2^, sheath gas temperature of 325°C, sheath gas flow rate of 12 L/min, capillary at 2,000 V, and nozzle at 500 V. The detector delta EMV(−) was 200. The QqQ data were preprocessed with Agilent MassHunter Workstation QqQ quantitative analysis software (version B0700). Each metabolite abundance level in each sample was divided by the median of all abundance levels across all samples for proper comparisons, statistical analyses, and visualizations among metabolites.

## References

[1] van Seventer JM, Hamer DH. 2016. Foodborne diseases, p 163–173. *In* Cockerham WC (ed), International encyclopedia of public health, 2nd ed. Academic Press, San Diego, CA.

[2] Bartsch SM, Lopman BA, Ozawa S, Hall AJ, Lee BY. 2016. Global economic burden of norovirus gastroenteritis. PLoS One 11:e0151219. doi:10.1371/journal.pone.0151219. 27115736 PMC4846012

[3] Ettayebi K, Crawford SE, Murakami K, Broughman JR, Karandikar U, Tenge VR, Neill FH, Blutt SE, Zeng X-L, Qu L, Kou B, Opekun AR, Burrin D, Graham DY, Ramani S, Atmar RL, Estes MK. 2016. Replication of human noroviruses in stem cell-derived human enteroids. Science 353:1387–1393. doi:10.1126/science.aaf5211. 27562956 PMC5305121

[4] Jones MK, Watanabe M, Zhu S, Graves CL, Keyes LR, Grau KR, Gonzalez-Hernandez MB, Iovine NM, Wobus CE, Vinjé J, Tibbetts SA, Wallet SM, Karst SM. 2014. Enteric bacteria promote human and mouse norovirus infection of B cells. Science 346:755–759. doi:10.1126/science.1257147. 25378626 PMC4401463

[5] Nordgren J, Svensson L. 2019. Genetic susceptibility to human norovirus infection: an update. Viruses 11:226. doi:10.3390/v11030226. PMC6466115 30845670

[6] Karandikar UC, Crawford SE, Ajami NJ, Murakami K, Kou B, Ettayebi K, Papanicolaou GA, Jongwutiwes U, Perales MA, Shia J, Mercer D, Finegold MJ, Vinjé J, Atmar RL, Estes MK. 2016. Detection of human norovirus in intestinal biopsies from immunocompromised transplant patients. J Gen Virol 97:2291–2300. doi:10.1099/jgv.0.000545. 27412790 PMC5756488

[7] Bok K, Parra GI, Mitra T, Abente E, Shaver CK, Boon D, Engle R, Yu C, Kapikian AZ, Sosnovtsev SV, Purcell RH, Green KY. 2011. Chimpanzees as an animal model for human norovirus infection and vaccine development. Proc Natl Acad Sci USA 108:325–330. doi:10.1073/pnas.1014577107. 21173246 PMC3017165

[8] Van Dycke J, Ny A, Conceição-Neto N, Maes J, Hosmillo M, Cuvry A, Goodfellow I, Nogueira TC, Verbeken E, Matthijnssens J, De Witte P, Neyts J, Rocha-Pereira J. 2019. A robust human norovirus replication model in zebrafish larvae. PLoS Pathog 15:e1008009. doi:10.1371/journal.ppat.1008009. 31536612 PMC6752765

[9] Seo DJ, Jung D, Jung S, Ha S-K, Ha S-D, Choi I-S, Myoung J, Choi C. 2018. Experimental miniature piglet model for the infection of human norovirus GII. J Med Virol 90:655–662. doi:10.1002/jmv.24991. 29106738

[10] Woodward J, Gkrania-Klotsas E, Kumararatne D. 2017. Chronic norovirus infection and common variable immunodeficiency. Clin Exp Immunol 188:363–370. doi:10.1111/cei.12884. 27753065 PMC5422859

[11] Roth AN, Karst SM. 2016. Norovirus mechanisms of immune antagonism. Curr Opin Virol 16:24–30. doi:10.1016/j.coviro.2015.11.005. 26673810 PMC4821668

[12] Esposito S, Principi N. 2020. Norovirus vaccine: priorities for future research and development. Front Immunol 11:1383. doi:10.3389/fimmu.2020.01383. 32733458 PMC7358258

[13] Lee S, Liu H, Wilen CB, Sychev ZE, Desai C, Hykes BL, Jr, Orchard RC, McCune BT, Kim K-W, Nice TJ, Handley SA, Baldridge MT, Amarasinghe GK, Virgin HW. 2019. A secreted viral nonstructural protein determines intestinal norovirus pathogenesis. Cell Host Microbe 25:845–857.e5. doi:10.1016/j.chom.2019.04.005. 31130511 PMC6622463

[14] Kolawole AO, Rocha-Pereira J, Elftman MD, Neyts J, Wobus CE. 2016. Inhibition of human norovirus by a viral polymerase inhibitor in the B cell culture system and in the mouse model. Antiviral Res 132:46–49. doi:10.1016/j.antiviral.2016.05.011. 27210811 PMC4980194

[15] Hosmillo M, Chaudhry Y, Nayak K, Sorgeloos F, Koo BK, Merenda A, Lillestol R, Drumright L, Zilbauer M, Goodfellow I. 2020. Norovirus replication in human intestinal epithelial cells is restricted by the interferon-induced JAK/STAT signaling pathway and RNA polymerase II-mediated transcriptional responses. mBio 11:e00215-20. doi:10.1128/mBio.00215-20. 32184238 PMC7078467

[16] Kindler V, Zubler RH. 1997. Memory, but not naive, peripheral blood B lymphocytes differentiate into Ig-secreting cells after CD40 ligation and costimulation with IL-4 and the differentiation factors IL-2, IL-10, and IL-3. J Immunol 159:2085–2090. 9278293

[17] Song J, Li F, Leier A, Marquez-Lago TT, Akutsu T, Haffari G, Chou KC, Webb GI, Pike RN. 2018. PROSPERous: high-throughput prediction of substrate cleavage sites for 90 proteases with improved accuracy. Bioinformatics 34:684–687. doi:10.1093/bioinformatics/btx670. 29069280 PMC5860617

[18] Kronqvist N, Sarr M, Lindqvist A, Nordling K, Otikovs M, Venturi L, Pioselli B, Purhonen P, Landreh M, Biverstål H, Toleikis Z, Sjöberg L, Robinson CV, Pelizzi N, Jörnvall H, Hebert H, Jaudzems K, Curstedt T, Rising A, Johansson J. 2017. Efficient protein production inspired by how spiders make silk. Nat Commun 8:15504. doi:10.1038/ncomms15504. 28534479 PMC5457526

[19] Waters LR, Ahsan FM, Wolf DM, Shirihai O, Teitell MA. 2018. Initial B cell activation induces metabolic reprogramming and mitochondrial remodeling. iScience 5:99–109. doi:10.1016/j.isci.2018.07.005. 30240649 PMC6123864

[20] Caro-Maldonado A, Wang R, Nichols AG, Kuraoka M, Milasta S, Sun LD, Gavin AL, Abel ED, Kelsoe G, Green DR, Rathmell JC. 2014. Metabolic reprogramming is required for antibody production that is suppressed in anergic but exaggerated in chronically BAFF-exposed B cells. J Immunol 192:3626–3636. doi:10.4049/jimmunol.1302062. 24616478 PMC3984038

[21] Oda H, Kolawole AO, Mirabelli C, Wakabayashi H, Tanaka M, Yamauchi K, Abe F, Wobus CE. 2021. Antiviral effects of bovine lactoferrin on human norovirus. Biochem Cell Biol 99:166–172. doi:10.1139/bcb-2020-0035. 32348689

[22] Tangye SG, Liu YJ, Aversa G, Phillips JH, de Vries JE. 1998. Identification of functional human splenic memory B cells by expression of CD148 and CD27. J Exp Med 188:1691–1703. doi:10.1084/jem.188.9.1691. 9802981 PMC2212517

[23] Nothelfer K, Sansonetti PJ, Phalipon A. 2015. Pathogen manipulation of B cells: the best defence is a good offence. Nat Rev Microbiol 13:173–184. doi:10.1038/nrmicro3415. 25659322

[24] Rojas OL, Narváez CF, Greenberg HB, Angel J, Franco MA. 2008. Characterization of rotavirus specific B cells and their relation with serological memory. Virology 380:234–242. doi:10.1016/j.virol.2008.08.004. 18789807 PMC2582161

[25] Moir S, Ho J, Malaspina A, Wang W, DiPoto AC, O’Shea MA, Roby G, Kottilil S, Arthos J, Proschan MA, Chun T-W, Fauci AS. 2008. Evidence for HIV-associated B cell exhaustion in a dysfunctional memory B cell compartment in HIV-infected viremic individuals. J Exp Med 205:1797–1805. doi:10.1084/jem.20072683. 18625747 PMC2525604

[26] Lee S, Wilen CB, Orvedahl A, McCune BT, Kim K-W, Orchard RC, Peterson ST, Nice TJ, Baldridge MT, Virgin HW. 2017. Norovirus cell tropism is determined by combinatorial action of a viral non-structural protein and host cytokine. Cell Host Microbe 22:449–459.e4. doi:10.1016/j.chom.2017.08.021. 28966054 PMC5679710

[27] Schultze JL, Michalak S, Seamon MJ, Dranoff G, Jung K, Daley J, Delgado JC, Gribben JG, Nadler LM. 1997. CD40-activated human B cells: an alternative source of highly efficient antigen presenting cells to generate autologous antigen-specific T cells for adoptive immunotherapy. J Clin Invest 100:2757–2765. doi:10.1172/JCI119822. 9389740 PMC508480

[28] Taube S, Kolawole AO, Höhne M, Wilkinson JE, Handley SA, Perry JW, Thackray LB, Akkina R, Wobus CE. 2013. A mouse model for human norovirus. mBio 4:e00450-13. doi:10.1128/mBio.00450-13. 23860770 PMC3735125

